# A method of searching for related literature on protein structure analysis by considering a user's intention

**DOI:** 10.1186/1471-2105-16-S7-S4

**Published:** 2015-04-23

**Authors:** Azusa Ito, Takenao Ohkawa

**Affiliations:** 1 Graduate School of System Informatics, Kobe University, 1-1, Rokkodai, 657-8501 Kobe, Japan; 2 Graduate School of System Informatics, Kobe University, 1-1, Rokkodai, 657-8501 Kobe, Japan

**Keywords:** article-based retrieval, literature on protein structure analysis, ontology, user's intention

## Abstract

**Background:**

In recent years, with advances in techniques for protein structure analysis, the knowledge about protein structure and function has been published in a vast number of articles. A method to search for specific publications from such a large pool of articles is needed. In this paper, we propose a method to search for related articles on protein structure analysis by using an article itself as a query.

**Results:**

Each article is represented as a set of concepts in the proposed method. Then, by using similarities among concepts formulated from databases such as Gene Ontology, similarities between articles are evaluated. In this framework, the desired search results vary depending on the user's search intention because a variety of information is included in a single article. Therefore, the proposed method provides not only one input article (primary article) but also additional articles related to it as an input query to determine the search intention of the user, based on the relationship between two query articles. In other words, based on the concepts contained in the input article and additional articles, we actualize a relevant literature search that considers user intention by varying the degree of attention given to each concept and modifying the concept hierarchy graph.

**Conclusions:**

We performed an experiment to retrieve relevant papers from articles on protein structure analysis registered in the Protein Data Bank by using three query datasets. The experimental results yielded search results with better accuracy than when user intention was not considered, confirming the effectiveness of the proposed method.

## Background

Knowledge on protein structure and function has been published and accumulated on the internet in the form of protein structure analysis publications. With advances in research in recent years, the number of publications on protein structure analysis has increased rapidly, causing difficulties for users by impeding their search of desired papers from among available articles on the topic.

In this study, we propose a search method for relevant articles by using a paper that the user is currently reading as an input query to provide the next article that would be desirable for the user to view. In the proposed method, the concepts referred to by the information on protein structure and function described in a paper are associated with the article. Thus, by regarding an article as a set of concepts, the degree of similarity between articles is calculated by the degree of similarity between sets of concepts. The similarity between concepts is evaluated by using a concept hierarchy graph obtained from existing databases.

In this framework, it should be noted that various information from a variety of perspectives is included in an article. For example, in relation to a paper that describes the results of analyzing the structure of a particular protein, a user may be interested in the molecular function of that protein or in another protein from the same family. Therefore, when the paper itself is used as a query, it is not possible to determine the perspective from which the user is seeking related articles, resulting in search results that may not be focused. At the same time, information obtained from only one paper is rarely sufficient when actually reading articles, and it is common for a user to read two or more related papers.

Therefore, by focusing on the fact that when searching the literature, relevant articles can be selected more easily based on past literature browsing history, not only the input article but two papers--the input article (primary article) and a related additional article--are employed as the input query. Furthermore, we assume that the intention of the user potentially resides in elements common to the input article and the additional article. Thus, by adjusting the degree of attention given to each concept that constitutes the concept hierarchy graph and modifying this graph in accordance with the input query, we are able to realize a relevant literature search that considers user intention.

Recently, various retrieval tools or services for scientific research articles have been provided[1-4], which are very useful and are widely used. In most of them, the citation-based approach, in which the relation between articles is estimated mainly using citation information, is used. Therefore, very newly published articles or the articles cited from few articles, are hard to be retrieved as related articles. On the other hand, many content-based methods for retrieving related documents from the query document have been proposed[5-9]. Similar to our proposed method, most of them evaluate the similarity between documents by regarding a document as a set of concepts on the concept hierarchy graph. However, in these methods, identification of the user intention based on more than one query articles has not been discussed. Focusing on the user intention by calculating the degree of attention is the important and original feature of our method.

## Methods

### Databases relevant to protein structure analysis

The literature targeted for search is represented by the set of articles referenced by each entry in the Protein Data Bank (PDB) [10]. In this study, we treat the in-formation described in an article on protein structure analysis, such as that on structure and function, as a set of concepts. From the relationships between these concepts associated with the articles on protein structure analysis, we consider calculating the relationship between papers. At this point, a variety of information is required, such as the structure and function of proteins, as well as biological terms. To obtain this information, the following databases are used in coordination.

• MEDLINE / PubMed [11]

• Protein Data Bank [10]

• SIFTS [12]

• Gene Ontology [13]

• InterPro [14]

**Figure 1:**
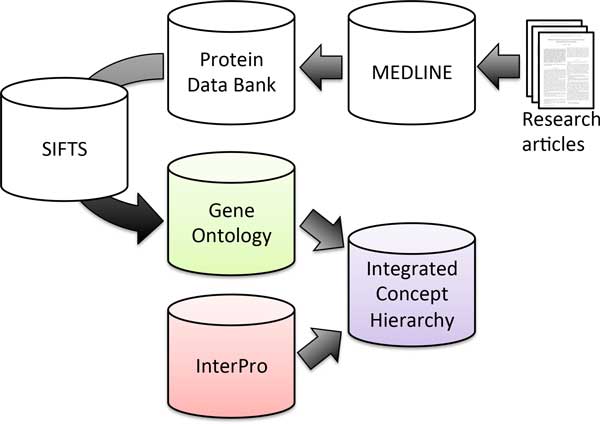
**Coordinated use of databases**. An article is converted to a set of concepts on the concept hierarchy by coordinated use of databases.

The method for coordinating the databases and using them to convert an article to a set of concepts is shown in Figure 1. Literature information was obtained from MEDLINE, a biomedical literature database in which each paper is assigned a PubMedID (PMID) number. In addition, for papers on protein structure analysis that are registered on MEDLINE, the protein structure analysis results are registered in the PDB. Furthermore, by using SIFTS, provided by UniProt [15], to connect the data for each protein registered in the PDB to Gene Ontology (GO)--a database that unifies the descriptions of biological concepts--the entire data can be handled as a set of concepts. Through these processes, an article on protein structure analysis can be considered as a set of concepts.

**Figure 2:**
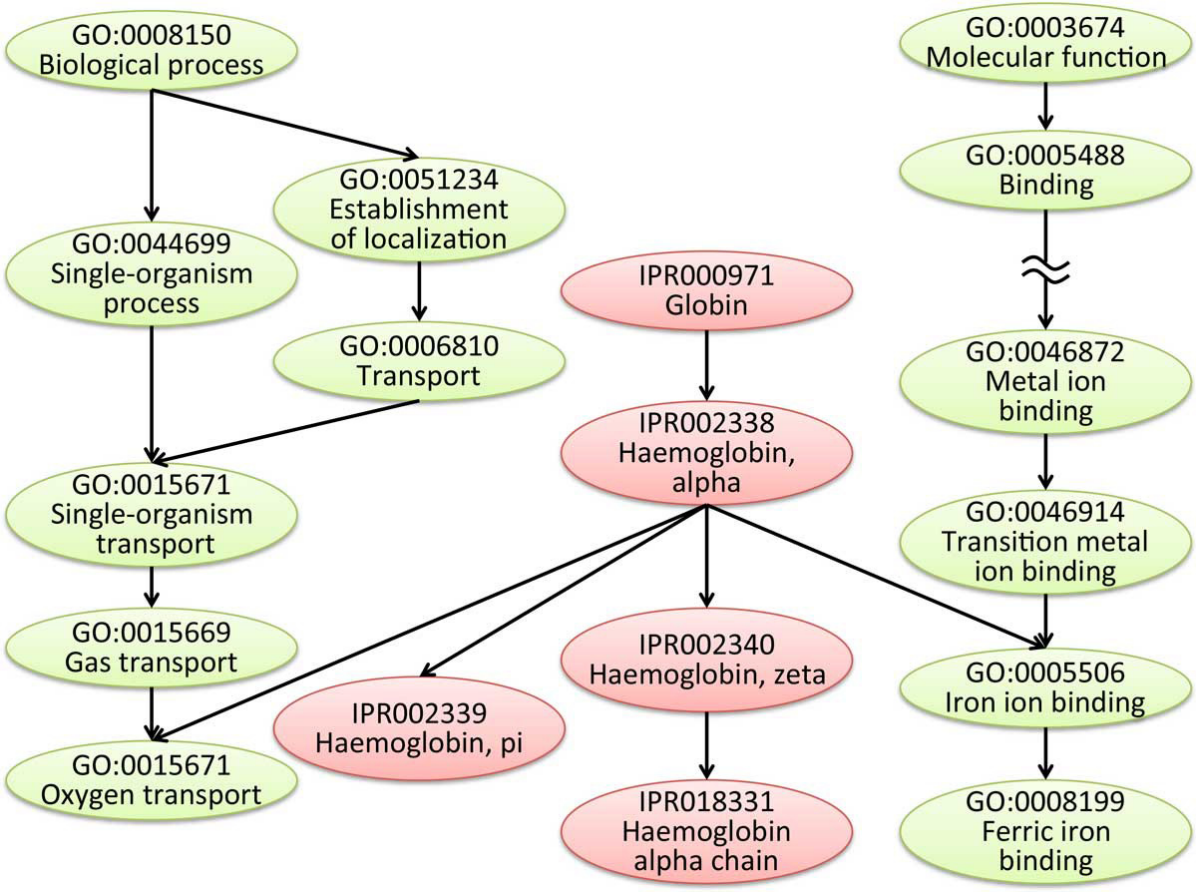
**Integration of GO and InterPro**. The directed acyclic graph shows an example of integration of GO (gray nodes) and InterPro (white nodes) constructed by using InterPro2GO cross-references.

All biological terms registered in the GO database belong to one of three categories: biological processes, molecular function, or cellular components. On the basis of these categories, three directed acyclic graphs with nodes consisting of the concepts of the registered biological terms are constructed. Similarly, InterPro is a database that defines hierarchical relationships between concepts. This database organizes and provides information relevant to protein structure analysis from the perspective of protein family, domain, functional site, etc. Whereas GO is a general biological concepts database, InterPro is a protein function database; however, because each can be used to construct a directed acyclic graph containing both superordinate and subordinate concepts, GO and InterPro graphs can be integrated when considering their use in the limited context of searching for articles related to protein structure analysis. This makes it possible to construct a directed acyclic graph that includes a wide variety of concepts. Figure 2 shows an example of GO and InterPro integration. Integration of GO and Inter-Pro is performed by using InterPro2GO mapping[16], which is the manually generated cross-references between InterPro and GO. Namely, if InterPro entry *i *is associated with GO term *t *in InterPro2GO mapping database, the link '*i → t*' is generated in the integrated concept hierarchy.

### Overview of the search method for relevant literature

**Figure 3:**
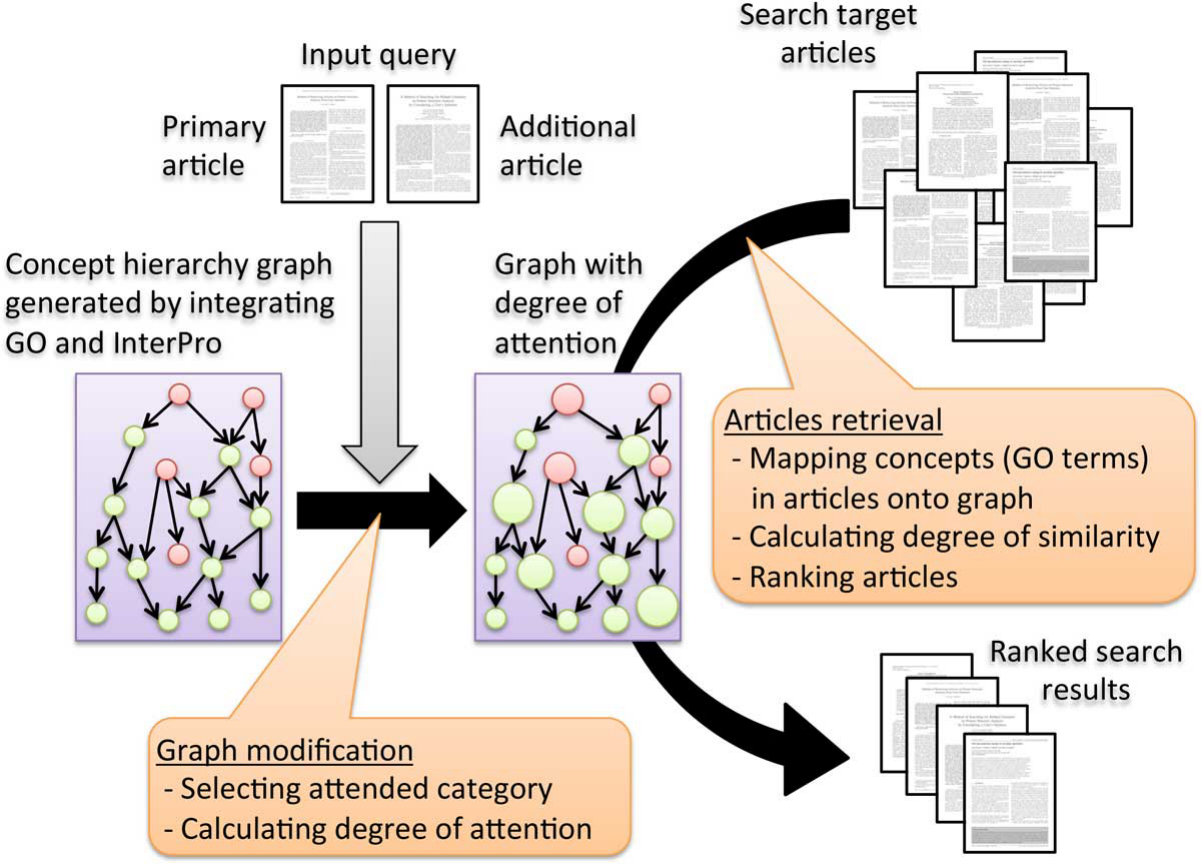
**Overview of the proposed method**. The input query consists of two articles, a primary article and an additional article. The concept hierarchy graph is modified by calculating the degree of attention by using two input articles. The similarity between the input article and each of search target articles is evaluated based on the modified graph.

Figure 3 shows an overview of the relevant literature search method proposed in this paper. This method uses an article as an input query (hereafter referred to as the input article). Then, as described above, the input article and the paper targeted for search are each converted into sets of concepts through the coordinated use of multiple databases, and the relationship between the two sets of concepts are determined to evaluate the relationship between articles.

The nodes of parent-child relationships in a concept hierarchy graph are constructed such that the concepts of parents are abstractions of those of their children and the concepts of children are specific examples of those of their parents. In other words, two concepts that are positioned closely can be regarded as similar. By using this property, the degree of similarity between two concepts is evaluated based on their positional distance in the graph. By using the set of concepts obtained from both the input article and search target article, the degree of similarity between all concepts is determined. The relationship between the input article and search target article is evaluated by using the sum of these similarities. At this point, when the input query consists of only one paper, it is difficult to determine the perspective from which the user seeks related articles.

Therefore, rather than using only a single input query, an additional paper related to articles the user seeks is added to the input query, which results in the establishment of a search method that reflects a user's search intention. The additional paper used as input is termed 'additional article.' At first glance, the task of selecting a relevant paper and adding it to the query may appear to impose difficult demands upon the user. However, during an actual database search of articles, it is often the case that a user has, in advance, browsed several papers related to the topic being searched. Considering that such information can be obtained from the search and browse history, it is likely that the addition of a relevant article as an input query can easily be performed by a user.

In order to achieve a search operation that reflects a user's intention, the perspective of interest is estimated from both the input article (primary article) and additional article, and the appropriate level of attention is assigned to each concept. To estimate the perspective of interest, the conceptual hierarchy graph is used as in calculating the degree of similarity. Analyses are carried out to determine which of the seven categories to which the concepts of the concept hierarchy graph belong is attended to by the user. Then, among the concepts that belong to the category estimated to represent the user's interest, those with strong connections to both the input article and additional article are assigned a degree of attention, based on which the degree of similarity is then varied. Thus, a relevant literature search that reflects the intention of the user is realized.

### Calculation of the degree of similarity between concepts

In order to evaluate the degree of similarity between two concepts, we use a common superordinate concept on the conceptual hierarchy graph that integrates GO and InterPro databases. A common ancestor of two concepts is defined as a concept that best encompasses both concepts; therefore, the similarity between the two decreases as each concept becomes more separated from its common ancestor. The lowest common superordinate concept is determined as follows.

**Definition 1 The lowest common superordinate concept for two concepts in the concept hierarchy graph**. *Let S***(***t***) ***be a set that eliminates the three concepts at the root of directed acyclic graphs in GO from the set of common ancestors of concept t in the concept hierarchy graph G, and T_onSP _***(***a, b***) ***be the set of nodes along the shortest path from ancestor b of concept (node) a to a (The node set T_onSP _***(***a, b***) ***includes both nodes a and b). Then, × ***∈ ***S***(***t*_1_**) ***∩ S***(***t*_2_**)***, which satisfies $\underset{x\in S\left(t_{1}\right)\cap S\left(t_{2}\right)}{\text{argmin}}$ as the lowest common superordinate concept of the two concepts t*_1 _*and t*_2_.

**Definition 2 The shortest-related path pair on the concept hierarchy graph**. *When the lowest common superordinate concept of two concepts (nodes) t*_1 _*and t*_2 _*is defined as t_lca_, the pair of paths from t_lca _to t*_1 _*and from t_lca _to t*_2 _*is called the shortest-related path pair of t*_1 _*and t*_2_.

The similarity between concepts is calculated by using the shortest-related path pair as introduced in Definition 2. If there are several shortest-related path pairs, one arbitrary path pair is selected from among them. As noted above, two concepts can be regarded as more similar the fewer the concepts that are located on the path pair. Therefore, from the concepts that constitute the shortest-related path pair between concepts, the degree of similarity between concepts *Sim^concept^*(*t*_1_*, t*_2_) is defined as follows.

**Definition 3 Degree of similarity between concepts**. *The degree of similarity between two concepts t*_1 _*and t*_2_*, Sim^concept^*(*t*_1_*, t*_2_) *is defined by the equation below*.

(1) $$\begin{array}{c} Sim^{concept}\left(t_{1},t_{2}\right)\\ =\left\{\begin{array}{c} \frac{1}{\underset{t\in T_{onSP\left(t_{1},t_{lca}\right)}\cup T_{omSP\left(t_{2},t_{lca}\right)}}{\prod }\alpha^{\left(1-att\left(t\right)\right)}}\\ \;\text{if there is a common superordinate}\\ \;\text{concept of }t_{\text{1}}\;\text{and}\;t_{\text{2}}\\ 0\\ \;\text{otherwise} \end{array}\right) \end{array}$$

In this equation, *att*(*t*) is the degree of attention that represents the extent to which a user attends to node *t*, and is assumed to take a value of 0 *≤ att*(*t*) *≤ *1. In general, the degree of attention is 0, but for a concept estimated to be deemed important under the user's search intention, the value is larger than 0. The method for calculating the degree of attention is described later. Furthermore, *α *is a constant parameter, and as the degree of similarity decreases as the sum of the number of nodes that exist in the paths from the lowest common superordinate concept of two concepts to each concept becomes larger, *α *must be greater than 1. Similar to this study, Done *et al.* conducted research on annotation using the hierarchical structure of GO to evaluate the similarity between concepts based on the path length of the hierarchical graph [17]. There it was assumed that when the number of nodes is 8, which is half the maximum depth from the GO root to the terminal, the degree of similarity between concepts is approximately 1%. Accordingly, *α *= 1.7, the value that gives *α*^*−*8 ^≃ 0.01, is used in our method.

Since lineages of GO and InterPro have been constructed from the different level nodes, the evaluation of the similarity based on the path length (the number of edges) in the integrated concept hierarchy is not exactly appropriate. The normalization of the level by giving the weight values to the edges is one of the remaining works.

### Estimation of the attention category of users by using an additional article

We propose to identify the search intention of a user from the relationship between the set of concepts assigned to the input article and that assigned to the additional article. We estimate the user's perspective of interest from the category of the lowest common superordinate concept for the concept sets of the input article and additional article within the concept hierarchy graph. Furthermore, based on this perspective, we calculate the degrees of attention for the concepts attended to by the user.

GO concepts belong to one of the following categories: biological processes, molecular function, or cellular components. InterPro concepts belong to one of the following four categories: family, domain, repeat, or site. Accordingly, the concepts in the concept hierarchy graph that integrate GO and InterPro belong to one of seven total categories.

In estimating the category attended to by the user, the category of the lowest common superordinate concept is obtained for all combinations of the shortest-related path pairs between the input article and additional article. Because the lowest common concept is the most specific concept that encompasses the elements shared by the concepts, we may infer that a category with many lowest common concepts reflects the user's perspective. However, the category to which the common superordinate concept belongs is highly dependent on the category of concepts assigned to the input article and additional article as an input query. This is because the concept hierarchy graph is configured in a form that interconnects the nodes of the three unconsolidated directed acyclic graphs that comprise GO, by utilizing the superordinate-subordinate relationships in InterPro. From this, in the shortest-related path pairs between concepts that belong to different categories, the lowest common superordinate concept will always be contained within InterPro; hence, the tendency of the common superordinate concept to belong to an InterPro category is strong. On the other hand, the common superordinate concept of two concepts that belong to the same category tends to belong to the same category with regard to both concepts. In addition to considering such tendencies, it is important to note that there are large biases in the number of concepts that belong to each InterPro category. By correcting for this bias, the category attended to by the user is defined as follows.

**Definition 4 Attended category**. *Let T_ALL _be the entire set of concepts that appears in the concept hierarchy graph; T_p _the set of concepts in the input article (primary article); Ta the set of concepts in the additional article; T_lca _the set of lowest common superordinate concepts that reflects the total combination of the set of concepts in the input article and additional article; |T | the number of concepts contained within the set of concepts T; and n*(*T, C*) *the number of concepts that belong to category C inside the set of concepts T. In addition, let ***C_Go _***be the category contained within GO, and ***C_Ipr _***the category contained within InterPro. Then the correction value rev^C ^, which considers the characteristics of the concept hierarchy graph and the number of concepts registered by the InterPro category, is defined by the following formula, where the attended category is that for which the value of n*(*T_lca_, C*)*/rev^C ^is largest*.

(2) $$\begin{array}{c} rev^{C}\\ =\left\{\begin{array}{c} n\left(T_{p},C\right)n\left(T_{a},C\right)\\ \;\;\text{if}\;C\;\in \;C_{Go}\\ \left\{|T_{p}||T_{\alpha }|-\underset{C^{\prime }\in C_{Go}}{\sum }n\left(T_{p},C^{\prime }\right)n\left(T_{a},C^{\prime }\right)\right\}\\ \;\;\;\;\;\times \frac{n\left(T_{ALL},C\right)}{\underset{C^{\prime \prime }\in {\text{C}}_{\text{Ipr}}}{\sum }n\left(T_{ALL},C^{\prime \prime }\right)}\\ \;\;\text{if}\;C\;\in \;{\text{C}}_{\text{Ipr}} \end{array}\right) \end{array}$$

### Calculation of the degree of attention

We determine the value of *att*(*t*), which is the degree to which the user attends to concept *t *on the shortest related path pair, based on the user's search intentions. Since the nodes that reflect the user's search intention consist of those that are associated with the set of concepts in both the input article and additional article, only the concepts on the shortest-related path pair are assigned values greater than 0 for the degree of attention, and the degree of attention for all other nodes is assigned a value of 0. Also, the lowest common super-ordinate concept of two concepts is the most detailed among all concepts common to the two, and this node must match the perspective to which the user is attending. Accordingly, the range of the nodes for which the degree of attention is updated is determined as follows.

**Definition 5 Range of giving degree of attention**. *Let T_C _be the set of concepts that belong to the target category C among concepts that appear in the concept hierarchy graph. The range of giving the degree of attention for the input article T_p _and the additional article T_a _is defined as*

(3) $$R^{att}\left(T_{p},T_{a}\right)=\underset{\begin{array}{c} t_{p}\in T_{p},t_{a}\in T_{a},\\ t_{lca}\in T_{lca}\left(t_{p},t_{a}\right) \end{array}}{\cup }\left\{\begin{array}{cc} T_{onSP}\left(t_{p},t_{lca}\right)\cup T_{onSP}\left(t_{a},t_{lca}\right) & \text{if}\;t_{lca}\in T_{C}\\ 0̸ & \text{otherwise} \end{array}\right)$$

Regarding the nodes for which the degree of attention is updated, it is also possible to consider that the closer a concept is to one that has been assigned to the input query, the stronger the relationship is between the concepts. From this, the degree of attention *att^node^*(*t, t_p_, t_a_*) to concept *t *∈ *R_att_*(*T_p_, T_a_*), which reflects the concepts contained in the input article *t_p _*and in the additional article *t_a_*, is defined by using the distance between the concepts as follows.

**Definition 6 Degree of attention calculated from the shortest related path pair between concepts of the input article and additional article**. *Let t_lca _be the lowest common superordinate concept of concepts a and b; and*$T_{onSP\bar{lca}}\left(a,t_{lca}\left(a,b\right)\right)$*be the set of nodes on the shortest path from t_lca_, which is the lowest common superordinate concept between a and b, to a, excluding t_lca_*(*a, b*)*. Then, the degree of attention to concept t *∈ *R_att_*(*T_p_, T_a_*)*, which comes from the concepts contained within the input and additional articles, t_p _and t_a _(att^node^*(*t, t_p_, t_a_*)*), is defined as follows*.

(4) $$\begin{array}{c} att^{node}\left(t,\;t_{p}\text{, }t_{a}\right)\\ =\left\{\begin{array}{c} \frac{1}{\alpha^{len}SP\left(t_{p},t\right)}\\ \;\;\text{if}\;t\;\in \;T_{onSP\bar{lca}}\left(t_{p},t_{lca}\left(t_{p},t_{a}\right)\right)\\ \frac{1}{\alpha^{len}SP\left(t_{a},t\right)}\\ \;\;\text{if}\;t\;\in \;T_{onSP\bar{lca}}\left(t_{a},t_{lca},\left(t_{p},t_{a}\right)\right)\\ \frac{1}{2}\left(\frac{1}{\alpha^{len}SP\left(t_{a},t\right)}+\frac{1}{\alpha^{len}SP\left(t_{a},t\right)}\right)\\ \;\;\text{if}\;t=t_{lca}\left(t_{p},t_{a}\right)\\ 0\\ \;\;\text{otherwise} \end{array}\right) \end{array}$$

*len_SP _*(*t*_1_*, t*_2_) is the length of the shortest path from *t*_2_, which is the ancestor of *t*_1_, to *t*_1_. Thus, it is important to note that the degree of attention to concept *t, att*(*t*), is not absolute, but is determined according to the concepts included in the input and additional articles. Furthermore, if the length of the shortest related path pair is defined as the sum of the lengths of the two paths formed by the shortest related path pair between concepts, we may consider that the shorter this length, the stronger the relationship is between the specific contents of the two nodes. Conversely, for a long path pair, we may infer that the nodes will only be related in a broad and ambiguous sense. Hence, there is a need to change the degree of attention according to the length of the shortest related path pair. Therefore, the degree of attention is corrected in accordance with the length of the shortest related path pair. In addition, the normalized degree of attention *att^norm^*(*t, t_p_, t_a_*), set to take values between 0 and 1, is defined as follows.

**Definition 7 Normalized degree of attention that considers the path length of the shortest related path pair**. *The normalized degree of attention to concept t, att_norm_*(*t, t_p_, t_a_*)*, calculated on the basis of concepts t_p _and t_a _contained within the input and additional articles, respectively, is defined as follows*.

(5) $$att^{norm}\left(t,t_{p},t_{a}\right)=\frac{att^{node}\left(t,t_{p},t_{a}\right)}{\underset{t_{i}\in P\left(t_{p},t_{a}\right)}{\sum }att^{node}\left(t_{i},t_{p},t_{a}\right)}$$

In general, the input query (i.e., both the input article and additional article) contain several concepts. Therefore, in the case where a concept is included in multiple shortest related path pairs between concepts, it will be assigned several degrees of attention. In this case, to account for the degrees of attention assigned by the shortest related path pairs between all concepts within the input query, the sum of the degrees of attention to concept *t *from the input article *T_p _*and the additional article *T_a _*is calculated according to the formula below.

(6) $$att^{all}\left(t,T_{p},T_{a}\right)=\underset{t_{i}\in T_{p}.t_{a}\in T_{a}}{\sum }att^{norm}\left(t,t_{p},t_{a}\right)$$

In the above formula, because *att^all^*(*t, T_p_, T_a_*) exceeds the range of 0 to 1, the degree of attention must be normalized to this range. The final degree of attention is defined by using the maximum value of *att^all^*(*t, T_p_, T_a_*), as follows.

**Definition 8 The degree of attention to a concept**. *The degree of attention att*(*t*) *to concept t is defined as follows*.

(7) $$att\left(t\right)=\frac{att^{all}\left(t,T_{p},T_{a}\right)}{\underset{t\in T_{ALL}}{\text{max}}att^{all}\left(t,T_{p},T_{a}\right)}$$

From this, all nodes contained on the shortest path between concepts with regard to the input and additional articles have a degree of attention that falls between 0 and 1.

### Calculating similarity between documents by using the similarity between concepts

Although the degree of similarity between concepts can be determined from (1), because an article comprises a set of many concepts, it is necessary to consider a method to calculate the degree of similarity for sets of multiple concepts simultaneously in order to calculate the degree of similarity between articles. The degree of similarity of a search target article to the input article is defined by using (1) as follows.

**Definition 9 Degree of similarity of *T*_2_ to a set of concepts *T*_1_**.

(8) $$\begin{array}{c} Sim^{concepts}\left(T_{1},T_{2}\right)\\ =\underset{t_{1}\in T_{1}}{\sum }\underset{t_{2}\in T_{2}}{\text{max}}Sim^{concept}\left(t_{1},t_{2}\right) \end{array}$$

As can be seen from the above equation, the sets of concepts *T*_1 _and *T*_2 _are not interchangeable. When actually calculating the similarity between articles, *T*_1 _corresponds to the input article (primary article) and *T*_2 _corresponds to the search target article. Articles highly relevant to the input documents are then defined as search target articles that contain many concepts highly similar to the set of concepts assigned to the input article, by calculating the degree of similarity of a concept in *T*_2 _that has the highest degree of similarity to each of the concepts in *T*_1_.

## Results and discussions

### Filtering searched articles

Because the articles targeted for search include all articles registered in the PDB, the number is enormous. This makes it difficult to complete similarity calculations within a practical amount of time. Therefore, the computation time is reduced by filtering the search target articles using keywords that indicate function or sequence similarity and clearly excluding unrelated papers from the search target articles[18].

### Evaluation of search results

In order to evaluate the accuracy of the results, it is desirable either to use correct data manually prepared by experts or to have experts judge whether articles ranked highly are related to the input query. However, because evaluations using these methods require a great deal of time and effort on the part of the experts, we used a more convenient method to prepare the correct data for evaluating the search results. This method identifies papers that cite both the input and additional articles (not only limited to papers on protein structure) and treats other articles on protein structure analysis that these papers cite as correct data. Namely, if there is an article *d_i _*that cites the references **R**(*d_i_*) = {*R*(*d_i_*)_1_*, R*(*d_i_*)_2_, ⋯, *R*(*d_i_*)*_n_*} in which both the input and the additional articles *T_p _*and *T_a _*are included, the articles in ∪*_i _***R**(*d_i_*) other than *T_p _*and *T_a _*compose correct data (positive data) for the query (*T_a_, T_p_*). The articles on protein structure analysis that have not been selected as the positive data are regarded as negative data.

The search results were evaluated by using mean average precision (MAP), a common measure for evaluating ranking methods based on the ranked outputs of search results and correct data[19]. The input queries used in the experiment are shown in Tables 1, 2, 3. One dataset consisted of 21 types of input query, which was constructed as combinations of the input articles (primary article) and additional articles. The dataset 1 is composed of three primary articles that are arbitrarily selected from collection of articles on protein structure analysis, and seven additional articles that are manually considered to have some relation to each of primary articles. The dataset 2 is constructed by exchanging the primary articles for the additional articles in the dataset 1. In the dataset 3, all of the primary articles and the additional articles are selected randomly. The numbers in the tables are the ID numbers assigned to each article in PubMed (PubMed ID).

**Table 1:** Input articles in dataset 1.

primary	additional	primary	additional	primary	additional
10558980	9497353	10966114	10558980	11961546	10558980

10558980	10966114	10966114	11099048	11961546	10966114

10558980	11591345	10966114	11591345	11961546	11099048

10558980	11853669	10966114	11961546	11961546	12553912

10558980	12535537	10966114	12535537	11961546	12820959

10558980	9261152	10966114	10350465	11961546	15537541

10558980	15660128	10966114	16365295	11961546	10205047

**Table 2:** Input articles in dataset 2.

primary	additional	primary	additional	primary	additional
9497353	10558980	10558980	10966114	10558980	11961546

10966114	10558980	11099048	10966114	10966114	11961546

11591345	10558980	11591345	10966114	11099048	11961546

11853669	10558980	11961546	10966114	12553912	11961546

12535537	10558980	12535537	10966114	12820959	11961546

9261152	10558980	10350465	10966114	15537541	11961546

15660128	10558980	16365295	10966114	10205047	11961546

**Table 3:** Input articles in dataset 3.

primary	additional	primary	additional	primary	additional
10700286	12121650	8611559	11727989	16083905	14572476

10700286	10467136	8611559	9174344	16083905	16873374

7966328	10562565	15327768	11707392	17070542	16406071

12297050	12297049	15327768	15327769	16740718	15507431

12297050	12620237	15294895	10504728	16740718	17718712

12517337	12086620	15294895	15070734	16732283	15931224

12517337	15274926	15126499	17038310	16732283	17643372

The key feature of the proposed method is that it obtains a search result that reflects the user's search intentions based on the relationship between the input and additional articles. In order to confirm the usefulness of this method, we also performed an experiment in which the additional article was not considered at all. A comparison between providing and not providing the additional article is shown in Table 4. In all datasets, we confirmed that considering the search intention by providing an additional article increased the MAP value. In particular, in the results from dataset 1, we obtained a significant difference in MAP values at a level of *P <*0.05 depending on whether the reflection of intention was present or absent based on the Wilcoxon signed-rank test[20].

**Table 4:** Mean Average Precision with or without additional article and with or without estimation of attended category.

	MAP with additional article		
**Dataset**	**with attended category estimation**	**without attended category estimation**	**MAP without additional article**

1	0.568	0.529	0.545

2	0.478	0.450	0.441

3	0.521	0.514	0.519

In this experiment, nodes that changed the degree of attention were selected by estimating the category that the user attended. To confirm the usefulness of this approach, we compared it with a method that assigns a degree of attention to all nodes contained within the shortest related path pairs without estimating the attended category of the user. The results are also shown in Table 4. In all datasets examined, MAP values increased by estimating the attended category. Regarding the results of dataset 1, we obtained a significant difference at a level of *P <*0.05 depending on whether or not the attended category was estimated, based on the Wilcoxon signed-rank test.

### Discussion on search results and correct data

In this study, for convenience, we prepared correct data based on citation information in order to evaluate the search results. However, by generating correct data using citation information, it is possible that articles similar to the input query were not included because the number of papers that cite new literature is less than the number of papers that cite old articles. Therefore, we discuss the four highest ranked articles with regard to the search results for input query (10558980, 9497353)--with an average precision value of 0.3073 and a particularly low evaluation in dataset 1. Table 5 shows the search results of input query (10558980, 9497353), which were arranged in the order of degree of similarity, as well as the results showing whether they matched with the correct data and the publication year of each paper. Articles 10558980 and 9497353 are commonly about ubiquitin enzymes, with the former and latter concerned with ubiquitin-conjugating enzyme and ubiquitin protein ligase, respectively. In article 10558980, a description of the structure and function of ubiquitin protein ligase is followed by a description of the structure of the HECT domain, which involves a ubiquitin-conjugating enzyme bound to ubiquitin protein ligase. In article 9497353, a description of the function of ubiquitin-conjugating enzymes is followed by a description of the protein degradation performed by their reciprocal action with ubiquitin protein ligase. At this point, only articles 10966114 and 11591345 were among the top four papers in the search results included in the correct data. However, articles 16307917 and 15931224, which were not chosen as correct data, also contain descriptions of the aforementioned ubiquitin enzymes. This suggests a high possibility that content related to that shared by articles 10558980 and 9497353 is described. Furthermore, examining the relationship between the publication year, correct data, and evaluated literature in Table 5 reveals that articles 15931224 and 16307917 are relatively new compared to articles 10966114 and 11591345, which are ranked highly and included as correct data. Therefore, it is quite possible that the former were not included in the correct data for this reason. These findings suggest that a search of relatively new relevant articles, which is normally difficult to perform only from citation information, is made possible by using the proposed method. In addition, the findings indicate the importance of producing more appropriate correct data to adequately evaluate the method.

**Table 5:** An example of highly ranked similar articles

rank	articles	included in correct set	publication year
1	10966114	yes	2000

2	15931224	no	2005

3	16307917	no	2005

4	11591345	yes	2001

## Conclusion

In this paper, by using an input article and an additional article as the input query, we proposed a method of implementing a relevant literature search that considers the user's intentions. The proposed method measured the relationship between articles by calculating the degree of similarity between concepts using a concept hierarchy graph. The method is characterized by its ability to reflect user intention in the search results by considering the category attended by the user and the degree of attention.

To confirm effectiveness of the proposed method, an experiment to evaluate a search of relevant papers among PDB-registered articles on protein structure analysis was performed. Results showed that by using the attended category and degree of attention to reflect user intention, search accuracy was confirmed to be improved. Furthermore, among the searched articles that were ranked highly by the proposed method, verification of the results on articles deemed to be incorrect data revealed that relatively new papers are unlikely to be included in the correct data, regardless of the similarity of content. This is because the correct data used in the evaluation experiment was generated from citation information for the purpose of convenience. These results emphasize the importance of establishing an absolute standard for correct data. In the future, to allow for more precise evaluations, appropriate construction of correct data is one of the major issues that will need to be addressed.

The method of evaluating similarity between articles proposed in this paper has been applied to the task of related articles retrieval, but has a potential of being applied to any other tasks e.g. articles classification, which is another remaining work.

## Competing interests

The authors declare that they have no competing interests.

## Authors' contributions

AI wrote the software, carried out the computational experiments, and drafted the manuscript. TO conceived of the study, and participated in its design and coordination and helped to draft the manuscript. All authors read and approved the final manuscript.

## Declarations

The publication costs for this article were funded by Grants-in-Aid for Scientific Research(B) 24300056, Japan Society for the Promotion of Science.

This article has been published as part of *BMC Bioinformatics *Volume 16 Supplement 7, 2015: Selected articles from The 11th Annual Biotechnology and Bioinformatics Symposium (BIOT-2014): Bioinformatics. The full contents of the supplement are available online at http://www.biomedcentral.com/bmcbioinformatics/supplements/16/S7.
